# Quick insights into whisky — investigating rapid and efficient methods for sensory evaluation and chemical analysis

**DOI:** 10.1007/s00216-023-04883-5

**Published:** 2023-09-02

**Authors:** Helen Haug, Andreas T. Grasskamp, Satnam Singh, Andrea Strube, Tilman Sauerwald

**Affiliations:** 1 Department of Sensory Analytics & Technologies, Fraunhofer Institute for Process Engineering and Packaging IVV, Giggenhauser Str. 35, 85354 Freising, Germany; 2 Chair of Aroma and Smell Research, Department of Chemistry and Pharmacy, Friedrich-Alexander-Universität Erlangen-Nürnberg, Henkestraße 9, 91054 Erlangen, Germany

**Keywords:** Whisky, Gas chromatography-mass spectrometry, Automated compound detection, Human sensory, Classification

## Abstract

The evaluation of aroma properties of beverages, both analytically as well as with human sensory studies, is a challenging task and most often related to time- and cost-intensive analyses. Whisky is a spirit offering a wide variety of aroma impressions caused by a complex mixture of aroma active compounds. In the present study, methods for the efficient evaluation of aroma characteristics are evaluated for 16 whisky samples of different origins (Scotch and American). Rate all that apply (RATA) was applied as a rapid method for the sensory evaluation of whiskies. Sensory evaluation of the samples led to the determination of eight significant aroma attributes: caramel-/cream caramel-/toffee-like, vanilla-like, (canned) peach-like, phenolic, smoky, fruity, flowery and (fermented) apple-/cider-like. Chemical analysis was conducted by stir bar sorptive extraction (SBSE) coupled to gas chromatography-mass spectrometry in combination with an in-house data processing tool for semi-automated analyte detection. Through chemical analysis of the whisky samples and automated compound detection, we report over 200 mostly aroma-active volatiles. To test both approaches for their potential for sample classification, a simple classification problem (Scotch vs. American) was applied. Linear discriminant analysis (LDA) indicates both that sensory evaluation by RATA (97.86%) and the applied analytical procedure (96.94%) are suitable for the distinction between the two whisky types. In both approaches, potential markers were determined for the classification. These investigations build a solid foundation for the implementation of a versatile platform facilitating rapid and efficient aroma evaluation in various foodstuffs and beverages.

**Supplementary Information:**

The online version contains supplementary material available at 10.1007/s00216-023-04883-5.

## Introduction

Whisky is a complex spirit offering a large variety of sensory experiences. Therefore, it is highly appreciated and popular among casual consumers and connoisseurs equally. The sensory characteristics of the beverage offer a huge spectrum, ranging from floral, fruity to phenolic and smoky notes [1, 2]. Many studies have so far focused on the characterization of whisky constituents, including aroma compounds [3–6]. Aroma active volatiles that have often been reported to be found in different kinds of whisky include 2-phenylethanol, 3-methylbutylacetate, furfural, *cis/trans* whisky lactone, vanillin, γ-nonalactone, and different phenolic compounds such as 4-ethylphenol and 4-methylphenol. Beyond that, carbonic acids such as octanoic and decanoic acid as well as different ethyl and isoamyl esters represent typical whisky compounds [4, 5, 7–10]. The aroma profile of whisky perceived through smelling or drinking the spirit results from a wide range of compounds in different concentrations and of different odor qualities [2, 4, 5]. As a whisky’s aroma is the most important feature of the beverage when it comes to consumer preference, it is crucial to monitor and maintain high sensory quality standards. Aroma analysis is a complex and versatile procedure that, depending on the scope of the analysis, includes various different steps. Generally, gas chromatography coupled to mass spectrometry (GC–MS) and an olfactory detection port (OPD) plays a major role for the analysis of volatile aroma compounds. To identify these compounds, mainly three factors are taken into account: the mass spectra, the linear retention indices (L-RI) as well as the odor quality detected at the ODP [5, 11, 12]. The comparison with reference compounds then leads to unequivocal identification of the aroma compounds. For whisky, as for other matrices, sample preparation plays a major role. Methods vary regarding the time and personnel required, the analytes that are extracted, and the usage of equipment and solvent [5, 7, 8]. Therefore, the sampling method has to be chosen carefully considering these aspects. Relating to aroma compound analysis in whisky, different methods for sample preparation and analyte extraction have been applied so far. This includes, for example, solvent extraction followed by the solvent-assisted flavor evaporation (SAFE) technique [5], (headspace) solid phase microextraction (SPME) [6–8], and stir bar sorptive extraction (SBSE) [8, 13, 14]. SBSE uses a sorbent-coated stir bar (e.g., with polydimethylsiloxane (PDMS)) which is placed into the liquid sample [15]. The analyte extraction is based on the partitioning between the sample and the sorbent. Baltussen et al. [15] proposed to estimate the partitioning of analytes between PDMS and water via its proportionality to the molecule’s octanol–water partitioning coefficient (K_O/W_), which is widely available for various different compounds.

For both, analytical as well as sensory investigations, data treatment plays an important role. Recent research shows that the use of analytical data on volatiles is a promising tool for the classification of different kinds of samples. This also applies for whisky, e.g., in relation to type, origin or label [9, 16] as well as authenticity [3, 17]. Thereby, different analytical methods can be applied as shown in the literature. These range from sophisticated GC analysis that offers the advantage of getting detailed information on chemical compositions to the use of sensor arrays in form of e-noses. The method of choice usually depends on the scope of the study and desired information content of the obtained analytical data. A comprehensive study for the classification of whisky samples based on GC–MS data was conducted by Stupak et al. [17]. A total of 191 whisky samples, including 71 malt whiskies, 77 blended Scotch whiskies, 20 samples previously identified as fake, and 23 additional malt whiskies, matured in more than one type of cask, were investigated. Comparing two different sampling strategies, headspace SPME and extraction with ethyl acetate, the latter proved more suitable to solve the classification problems. The samples were successfully classified regarding the cask used for maturing (Bourbon vs. Bourbon and wine cask) as well as regarding the type of whisky (blended, premium blended, and malt whiskies) and authenticity. Furthermore, marker compounds could be determined for some classifications [17]. González-Arjona et al. [18] investigated the use of four different pattern recognition procedures for the classification of 52 whisky samples (single malt Scotch, Bourbon, and Irish whisky) considering 28 compounds detected by GC–MS. The best results were obtained by artificial neural networks (multilayer perceptrons (MLP) and probabilistic neural networks (PNN)). Linear discriminant analysis (LDA) showed weaker performance for solving the classification problem as some class overlappings could be observed [18].

Apart from that, other analytical methods offering rapid or facilitated generation of data can be used to solve classification problems. Such methods might thereby not allow to obtain detailed information on single molecules responsible for the classification. Examples for alternative analytical strategies include the use of e-noses as shown by Zhang et al. [16] or direct analytical approaches without chromatographical separation as conducted by Wiśniewska et al. [19]. Even though the analytical approach is not directly comparable to the work presented in the present research, the handling of analytical data and use of processing methods show promising results for the classification of whisky samples.

In the same way as the development of more efficient analytical methods and corresponding data processing tools, the need for rapid sensory methods has risen and respective methods, such as rate all that apply (RATA) [20, 21] have been explored [2, 21–23]. As suggested within the present research paper, the use of chemometrics and statistical or even machine learning approaches might not only be advantageous for the classification of native sample properties, like origin, type, and authenticity. Moreover, such methods might also be promising regarding the combination of analytical and sensory data for the prediction or classification with respect to sensory parameters. This topic was treated within various research papers relating to different kinds of foodstuffs and beverages [24–26].

In the field of aroma research, the combination of instrumental and sensory studies plays a major role as the sensory impressions caused by single odorants or their combinations can hardly be determined instrumentally. A thorough investigation to reliably determine the full spectrum of aroma compounds or the key aroma compounds including quantitative methods requires GC–MS/O or even 2-dimensional GC–MS coupled to effortful analyte extraction methods, while reference standards are used for compound identification [5, 11, 27, 28]. A systematic and comprehensive investigation of aroma compounds and their impact on the overall aroma profile of a given sample (e.g., pastry [29], chocolate [30], alcoholic beverages [31, 32]) is conducted by applying the so-called sensomics approach. This includes a multi-step workflow for the characterization of aroma compounds applying instrumental as well as human sensory methods [33]. However, there is a need for fast or more efficient methods for some applications that require a reliable overview and high quality results on the aroma compounds present in the sample as well as the correlation to sensory impressions, while time and cost are the limiting factor. Nicolotti et al. [34], for example, applied a so- called sensomics-based expert system (SEBES) on wine and rum for the characterization of key aroma compounds using an approach based on artificial intelligence (AI). The use of a single analytical platform that does not necessitate human olfactory investigations was compared to the classical sensomics approach. The aim was to evaluate whether from a total of 226 known key food odorants, key aroma compounds of the samples could be predicted based on a limited number of analyses using software tools for compound identification, quantitation and calculation of odor activity values. Thereby, the authors concluded that key aroma compounds could be characterized based on only analytical data without the use of the human olfactory system by using their proposed platform with a good agreement between the two approaches (differences less than 20%) for most compounds [34]. Ashmore et al. [35] investigated the influence of diluting whisky samples on their sensory properties as well as the HS profile measured via HS-SPME GC–MS. The authors reported significant differences according to the dilution levels of the samples. Furthermore, they noticed some correlations between sensory attributes and the chemical profiling of the samples, suggesting that this could be a promising approach for predicting whisky aroma attributes [35].

The field of smart odor analysis, incorporating efficient or fast analytical methods as well as data science, therefore offers promising tools and opportunities not only for VOC analysis but also to gain further insights into large datasets, e.g., classification of aroma, provenience, and other quality aspects. For this reason, we are working on efficient VOC analysis methods for the evaluation of aroma and sensory properties for applications where such procedures are applicable and required. In this work, we present a platform for rapid evaluation and characterization of whisky characteristics by combining efficient and highly performant analytical and sensory evaluation methods. We demonstrate that this can be done based on automatically processed GC–MS data as well as sensory data with statistical methods to evaluate their potential to solve certain sample classification problems.

## Materials and methods

### Samples

For the sensory as well as chemical analysis 16 different commercially available whiskies were selected. These included 9 Scotch and 7 American whiskies of different types and origins (supplementary material, online resource Table S2). To adjust the alcohol content of the whiskies, we used a mineral drinking water (Acqua Panna, Nestlé Deutschland AG, Frankfurt, Germany). The alcohol content was adjusted to 40% (alcohol by volume, ABV) for chemical and sensory evaluation if the original alcohol content was not yet at 40% and for sensory analysis additionally to 20%. This resulted in one set of whiskies (16 samples at 40% ABV) for chemical analysis and 3 sets of samples for the sensory evaluation (16 samples at 40%, 16 samples at 20% ABV, and 8 whiskies at original alcohol content > 40%).

For this purpose, samples with higher alcohol contents than 40% were first diluted by adding the respective amount of drinking water to obtain a total of 170 ml using an Eppendorf pipette and measuring cylinder. For the sensory trials, all samples have additionally been diluted to an alcohol content of 20% vol. to gain further insights into the aroma properties of the samples. Thereby, a certain amount of whisky was filled up with drinking water to a total of 150 ml to obtain the respective ABV percentage of 20% vol. The respective samples are listed in Table S2 in the supplementary material (online resource Table S2).

Ten millilters of each sample was transferred into 40-mL amber glass bottles with screw cap and stored at room temperature for at least 24 h prior to sensory or chemical analysis to ensure comparability between samples and analyses.

### Chemicals and standards

For the determination of L-RI values after van den Dool and Kratz [36], a homologous series of n-alkanes C_6_-C_26_ (Sigma-Aldrich, Steinheim, Germany) was used. For the validation of the sampling procedure as well as the data analysis, two whisky-mimicking solutions (model whiskies) were prepared. The analytes were selected to represent a wide variety of compounds typically found in different kinds of whisky as reported in the literature [2, 4–6, 8, 9] as well as based on preliminary studies [37]. Therefore, the model whiskies contained typical whisky aroma compounds, covering a wide range of chemical functional groups and concentrations. Ethanolic solutions of reference compounds (online resources Table S1 for details on suppliers) were prepared. Defined volumes were mixed and filled up to 100 mL ethanol, and 2 mL of the ethanolic solution was then filled up with demineralized water to a total of 5 mL to obtain an alcohol content of 40% vol. For model whisky 1 and 2, the analytes presented in Table 1 were used, resulting in 27 single components in whisky model 1 and 26 single whisky components in model 2 (*cis* and *trans* whisky lactone counted as two) covering different compound classes, odorants and concentrations in both models. The concentrations were selected based on the typical concentration range of aroma compounds in whisky as reported by Poisson et al. [5].

**Table 1:** concentration of aroma compounds in model whiskies 1 and 2

Compound	CAS no	Concentration model whisky 1 [µg/mL]	Concentration model whisky 2 [µg/mL]
*β*-Damascenone ((*E*)-1-(2,6,6-trimethylcyclohexa-1,3-dien-1-yl)but-2-en-1-one)	23696-85-7	0.07	-
Furfural (furan-2-carbaldehyde)	98-01-1	3.07	1.53
3-Methylbutanal	590-86-3	0.36	0.68
2-Methylbutanal	96-17-3	0.23	0.09
2-Phenylethanol	60-12-8	13.90	8.90
2-Methyl-1-butanol	137-32-6	0.82	-
3-Methyl-1-butanol	123-51-3	9.39	46.93
3-Methylbutyl acetate	123-92-2	2.42	0.72
2-Phenylethyl acetate	103-45-7	2.47	4.02
3-Methylbutyl octanoate	2035-99-6	0.80	0.16
3-Methylbutyl decanoate	2306-91-4	-	0.31
Ethyl butanoate	105-54-4	0.30	0.30
Ethyl hexanoate	123-66-0	3.65	1.30
Ethyl heptanoate	106-30-9	0.39	-
Ethyl octanoate	106-32-1	3.23	2.02
Ethyl decanoate	110-38-3	2.12	2.76
Ethyl nonanoate	123-29-5	0.20	0.59
Ethyl tetradecanoate	124-06-1	0.13	0.21
Ethyl hexadecanoate	628-97-7	0.23	-
Octanoic acid	124-07-2	0.33	0.21
Decanoic acid	334-48-5	0.38	0.38
Dodecanoic acid	143-07-7	-	0.16
*cis/trans* Whisky-lactone^1^ (5-butyl-3-methyloxolan-2-one)	39212-23-2	0.36	0.36
γ-Nonalactone (5-pentyloxolan-2-one)	104-61-0	0.30	0.35
2-Methoxyphenol	90-05-1	3.36	0.54
Phenol	108-95-2	-	0.12
4-Methylphenol	106-44-5	0.03	0.05
4-Ethylphenol	123-07-9	0.02	-
4-Allyl-2-methoxyphenol, eugenol (2-methoxy-4-prop-2-enylphenol)	97-53-0	-	0.62
Vanillin (4-hydroxy-3-methoxybenzaldehyde)	121-33-5	0.36	1.43

^1^Counted as two compounds (*cis* and *trans* whisky lactone) separately, resulting in a total of 27 and 26 compounds for whisky model 1 and 2, respectively

Two internal standards were used for the comparison between samples, and for the validation of the detection tool as to the authors’ knowledge, those have not been reported as typical whisky compounds in the literature. 4-Chloro-2-methoxyphenol and *n-*undecane were mixed together in an ethanolic solution to obtain concentrations of 40–50 µg/mL (48.2 µg/mL and 43.25 µg/mL, respectively).

### Sensory evaluation of the samples

For the orthonasal sensory evaluation, the RATA method [20, 21] was used. For this purpose, applicable sensory attributes were selected by trained members of the Fraunhofer IVV sensory panel beforehand by reference to single whisky samples: solvent-like (nail polish-like), apple-like (including fermented apple/cider), pear-like, flowery, butter-like/butter-rum aroma, fruity, woody (freshly cut wood), honey-like, honeydew melon-like, caramel-like/cream caramel-like/toffee-like, peach-like (canned peach), coconut-like, spicy/clove-like, orange-like, smoky, phenolic, vanilla, and “others”. All 16 whisky samples were considered with alcohol contents of 20 and 40% vol. as well as their original alcohol content (if not at 40% vol.) resulting in a total of 40 samples. The sensory evaluation was performed by 11 volunteers (members of Fraunhofer IVV sensory panel; 8 female, 3 male, aged 26 to 43) at Fraunhofer IVV. Applying the RATA method, the whisky samples were evaluated within four sessions with 10 whisky samples each. The samples were coded with three-digit numbers and were served to the panellists in a randomized order. To ensure comparable procedure of the sensory evaluation, the panellists were instructed to transfer the 10 mL samples into tasting glasses and instantly close them with loose lids. The sensory evaluation was started after 10 min by smelling the sample first and indicating the most applicable attributes (max. 5) followed by an intensity rating for the selected attributes on a monadic scale of 1 (low intensity) to 3 (high intensity). Apart from the given attributes, the participants could also select “other” and qualitatively name further attributes (without intensity scaling) to indicate the appearance of attributes other than the ones listed. For the collection of sensory data, the Software program RedJade (RedJade Software Solutions LLC, Silicon Valley, US) was used.

### Sample preparation and analysis via gas chromatography-mass spectrometry

For the chemical analysis, all 16 whisky samples stored in 10 mL aliquots at 40% vol. alcohol content were considered. For the SBSE, 1 mL of each whisky sample or model whisky (40% vol.) was spiked with 100 µL of internal standard solution (*n*-undecane and 4-chloro-2-methoxyphenol), filled up to 10 mL with demineralized water, and 1.0 g of sodium chloride was added. The flask was shaken gently, and when all salt was dissolved, 2 mL of the sample were taken and transferred to a 10-mL glass vial with screw lid. A conditioned PDMS-coated Twister® with 1 cm length and 0.5 mm coating thickness (Gerstel GmbH & Co. KG, Mühlheim a. d. Ruhr, Germany) was added. Analytes were extracted subsequently by stirring with the Twister® for 1.5 h. After extraction, the Twisters® were dried with a lint-free tissue and stored in a closed GC-vial until analysis. For the two model whiskies, two blanks were analyzed by mixing 10 mL of distilled water each with 1 g of sodium chloride. Two milliliters were then each stirred with a Twister®. The same was done for the whisky samples; however, only two representative blank Twisters® were analyzed for all 16 whisky samples.

The Twisters® were transferred to thermal desorption (TD) glass tubes for analysis via GC–MS. A TD-Unit (TDU; Gerstel GmbH & Co. KG, Mühlheim a. d. Ruhr, Germany) was used for thermal desorption of the analytes from the Twisters®. The initial temperature was set to 40 °C with a solvent vent for 0.5 min and then raised to 280 °C at 120 °C/min; the final temperature was held for 8 min. The helium flow over the TDU was adjusted to 50 mL/min. Following the TD, the analytes were cryo-focused in a cooled injection system (CIS, Gerstel GmbH & Co. KG, Mühlheim a. d. Ruhr, Germany) at − 70 °C with liquid nitrogen. Desorption from the CIS was achieved by raising the temperature to 280 °C at 12 °C/s. The chromatographic separation within the GC system (Trace GC Ultra, Thermo Fisher Scientific GmbH, Dreieich, Germany) took place on a DB-FFAP column (30 m × 0.25 mm, film thickness 0.25 μm; J & W Scientific, Agilent Technologies, Waldbronn, Germany). The initial GC oven temperature was held at 40 °C for 2 min and then raised to 235 °C at 8 °C/min and held for 5 min. The analytes were transferred to the MS (DSQ II, Thermo Fisher Scientific GmbH, Dreieich, Germany) and detected with electron ionization (EI) at 70 eV in full-scan mode (m/z 35 – 399) with MS detection starting after 1 min.

For the analysis of the *n*-alkane reference [36], a solution of C_6_ to C_26_ in pentane (Th. Geyer, Renningen, Germany) was transferred into a micro vial within a TD tube and thermally desorbed (40 to 280 °C at 120 °C/min, holding time 5 min) and cryo-focused (− 50 °C). Concurrent with the Twister® analysis, the helium flow over the TDU was set to 50 mL/min, and the focusing trap was heated to 280 °C (12 °C/s) to transfer the analytes into the GC–MS system. The same chromatographic settings were applied as for sample analysis.

### Development and optimization of a semi-automated process

For the semi-automated processing of the GC–MS data obtained by the whisky analysis, a processing tool was used which we developed in-house previously as described in [38, 39] and shortly in the following section. The tool basically comprises two main steps: (i) raw data processing and (ii) compound detection including compound identity proposition.

#### Raw data processing

The processing of the raw GC–MS data includes the alkane reference as well as the sample analysis data. For this purpose, we developed a pre-processing pipeline for the examination of GC–MS data in .cdf format as described in detail in [39]. This includes the extraction of intensity matrices as number of scans × m/z followed by a peak deconvolution as applied by Biller and Biemann [40] for the alkane reference as well as sample data. For the processing of the alkane data, a procedure for alkane-peak detection was developed as described in detail in [39]. Based on this, the alkane chromatograms were analyzed to calculate the L-RIs of the compounds found in the sample data based on the equation by van den Dool and Kratz [36]. The procedure is described in our previous work [39] and includes methods for noise reduction and improved alkane detection.

#### General procedure for analyte detection

The raw data processing leads to the extraction of mass spectra and the determination of L-RIs for every peak detected in the chromatogram. Therefore, the second step aims at proposing analyte identities for the extracted peaks by comparison of mass spectra and L-RIs with appropriate databases. For this purpose, an internal database built with the AMDIS software (National Institute of Standards and Technology, NIST, Gaithersburg, USA) including mass spectra and L-RI of reference compounds was used. The database comprised around 700 mostly odor-active compounds with MS and L-RI characteristics and is constantly extended to ensure the covering of relevant whisky compounds. Apart from aroma active volatile compounds typically found in whisky and other foodstuffs, the database also contained entries for compounds that do not naturally occur in foods or can predominantly be found in non-food samples. As previously described [38, 39], the comparison of mass spectra was achieved by calculating the cosine similarity [39, 41] between the unknown mass spectrum of the analyte peak and the database mass spectra (Eq. 1).

1 $$scor{e}_{MS}=\frac{{\sum }_{i=1}^{n}M{S}_{lib,i}\times M{S}_{temp,i}}{\sqrt{{\sum }_{i=1}^{n}{\left(M{S}_{lib,i}\right)}^{2}}*\sqrt{{\sum }_{i=1}^{n}{\left(M{S}_{temp,i}\right)}^{2}}}$$

In Eq. 1, *n* is the continuous number of even m/z-values (ranging from 1 to an all-inclusive maximum of 5000) in the current mass spectrum *MS*_*temp*_ and the currently evaluated mass spectrum in the library *MS*_*lib*_. This score was calculated for all possible pairs of library entries and unidentified mass spectra as shown in [38, 39].

2 $$scor{e}_{MS+RI}=1-\frac{\left(1-scor{e}_{MS}\right)+abs\left(\frac{R{I}_{temp}-R{I}_{lib}}{R{I}_{lib}}\right)}{2}$$

In Eq. 2, we defined a combination of differences in L-RI and MS match quality by setting the *score*_*MS*_ calculated in Eq. 1 against the difference between the L-RI in the library *RI*_*lib*_ and the L-RI calculated for the unidentified mass spectrum *RI*_*temp*_. The obtained score_MS_ was further offset with the RI-match to another score (score_MS+RI_), as shown in Eq. 2. To ensure that only database entries with L-RI variances of + / − 30 compared to the peak under consideration were taken into account, a Heaviside function was multiplied with the score as shown in Eq. 3 and described in [38, 39].

3 $$f\left(\left|RI_{temp}-RI_{lib}\right|\right)=\left\{\begin{array}{c}0,\;if\;\left|RI_{temp}-RI_{lib}\right|>30\\1,\;if\;\left|RI_{temp}-RI_{lib}\right|\leq30\end{array}\right.$$

The resulting scores are thereby found to be between 0 (no match) and 1 (absolute match). For the compound detection and the final compound list, the minimum score for the proposition of an analyte’s identity can be chosen freely (e.g., 0.8). All unknown mass spectra (peaks) of the sample chromatograms went through this procedure.

#### Compound determination in model whisky and samples

For the qualitative determination of compounds in the model whiskies, one blank sample per model was considered. Thereby, analytes were detected  in the blank chromatograms (blank Twister® without model whisky extraction) as well as in the Twisters® used for analyte extraction from the model whiskies with a threshold score of 0.8 for compound detection (cf. Equation 2). Subsequently, all compounds that were noted in both the blank and model whisky Twisters® showing peak area ratios (peak area in model whisky/peak area in blank) smaller than 3 were subtracted from the model whisky compound list.

The 16 sample chromatograms were processed following the above-described procedure for compound detection applying a threshold of 0.8 with no blank subtraction.

### Statistical data evaluation

For the statistical evaluation of the sensory data, XLSTAT (Excel, Microsoft Cooperation, Redmond, USA) as well as MATLAB (R2021a, The MathWorks Inc., Natick, USA) was used. For the statistical analysis of the raw sensory data, it was split into qualitative (according to “Check all that apply,” CATA, data) and intensity (RATA) data and processed separately similar to the studies published by Vidal et al. [21]. The CATA data was treated with the “CATA data analysis tool” in XLSTAT (Excel, Microsoft Cooperation, Redmond, USA).

The statistical analysis of analytical data and the correlation of sensory and analytical data was conducted in MATLAB (The MathWorks Inc., Natick, USA). For the task of classifying whisky samples by type (Scotch or American), we used three non-exclusive sets of sensory and analytical data: (1) a combination of all RATA data (sum of ratings divided by participant counts) for 40% and 20% ABV for all 17 sensory attributes; (2) a list of relative peak areas (quantified by their abundance relative to 4-chloro-2-methoxyphenol) over all molecules detected in each whisky sample; and (3) a combination of (1) and (2). We then used each dataset in 5000 repetitions of training and testing a linear discriminant analysis (LDA) approach. Each of the repetitions featured one of 560 different, randomly drawn subsample sets of the full set of 16 samples, split into 13 samples for training, and 3 for testing. During each training repetition, a five-fold cross-validation was performed. There are 560 possible subsets of 13 in a full set of 16 $\left(\frac{16!}{13!*3!}\right)$ so a number of 5000 repetitions ensures for each set to occur. For each repetition, we calculated the accuracy of the cross-validated training as well as the test accuracy. The final percentage of correct classifications was then calculated as number of repetitions in which test accuracy was 100%, divided by number of repetitions.

## Results and discussion

### Sensory evaluation

#### Assessment of the parameters applied for the sensory evaluation

Corresponding to the goal of a fast and informative procedure for the analytical and sensory evaluation of whisky, a rapid sensory method using the RATA principle [20, 21] was chosen for the sensory testing. Within this trial, 16 different whiskies with different alcohol contents, resulting in a total of 40 samples, were evaluated orthonasally by 11 panellists within 4 sessions each. Therefore, the evaluation of 40 samples in total took around 2 h for each participant or 22 participant hours in total.

In order to obtain insightful information and to work out differences and similarities in the aroma characteristics of different whiskies by the RATA method, the selection of appropriate attributes for the sample description is of utmost importance. Within the sensory evaluations, all attributes have been used to describe the samples. The most often used attribute was fruity (156 times), while the attribute coconut-like was selected the least (29 times). This shows that all attributes were applicable to some of the samples and that the extent of their use differed. The option to state “other” attributes was used 33 times by 9 panellists. However, no statistical relevance could be detected between the attributes and the samples they were used for. Specifically, 9 of those attribute mentions (27.3%) came from one participant, and 51.5% of all alternative mentions came from just 3 participants. Taken together, these findings show that the selection of attributes was appropriate for the sensory description of the present whisky samples.

#### Qualitative sensory data

**Fig. 1:**
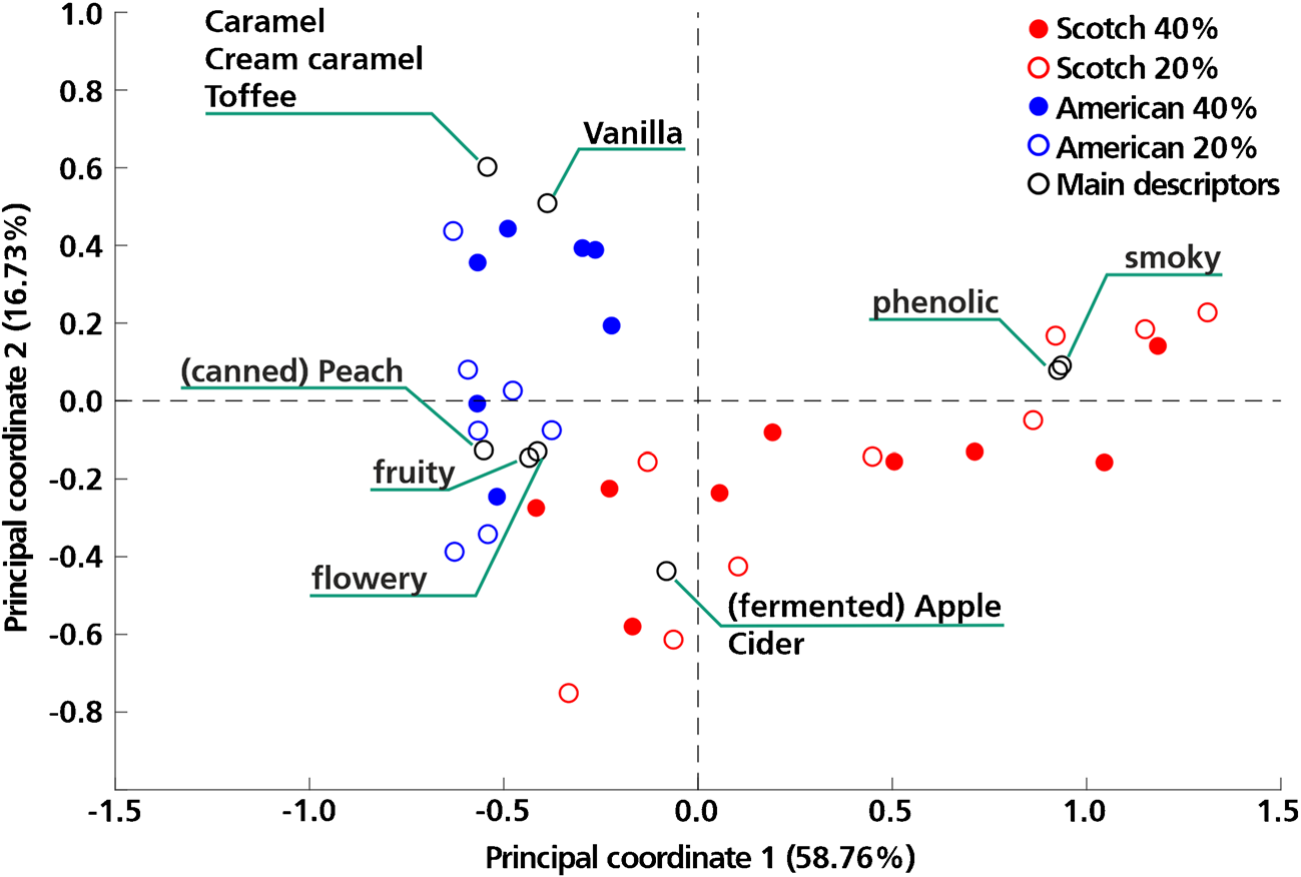
Sensory mapping of 16 whisky (9 Scotch, red and 7 American, blue) samples at two different ABV levels (20%, dot and 40%, circle) based on the evaluation of significant sensory attributes (main descriptors, black circles)

For the evaluation of sensory data, the whisky samples containing 20% and 40% ABV were considered. Data obtained by sensory evaluation was treated as described above (cf. “statistical data evaluation”). The qualitative sensory data obtained by applying the RATA-method corresponds to check-all-that-apply (CATA) data and was used for the mapping of the whisky samples based on their perceived aroma characteristics. Following statistical data processing including the determination of significant attributes for the sensory evaluation and principal coordinate analysis (PCoA), the whisky samples (20% and 40% ABV) could be mapped together with their descriptors. Caramel/cream caramel/toffee-like, vanilla-like, (canned) peach-like and (fermented) apple/cider-like were identified as significant attributes. PCoA and mapping of the Scotch and American whisky samples allowed the representation of the main characteristics of the samples within a sensory map. Principal coordinates (PCo) 1 and 2 represent ca. 75% of distance within the sample set, and therefore, the major differences can be accounted for by PCo 1 and 2. As can be seen in Fig. 1, the two sample groups (Scotch and American) occupy different parts of the map and can therefore be distinguished based on their position within the plot. However, there is still a significant intergroup variation within the two sample sets, which makes the differentiation more difficult. This variance, for example, becomes obvious for the attributes phenolic and smoky. These features are characteristic for Scotch and not for American whiskies; however, they do not apply to each of the Scotch samples.

Several studies show that the dilution of whisky has an impact on its sensory perception. Ashmore et al. [35], for example, showed that the sensory impressions of different whisky styles change when the beverage is diluted with water. According to the authors, differences between most styles become less detectable beyond a dilution ratio of 80% whisky/20% water, except for differences between American and Scotch whisky [35]. Karlsson and Friedman [42] also investigated the influence of dilution of whiskies on a molecular level focusing on guaiacol as an amphipathic compound. They found that depending on the alcohol content, the molecule can either be found rather at the water–air interface or surrounded by ethanol [42] with its obvious impact on partition coefficient and aroma release. Therefore, in our study, we also investigated whether a significant effect on classification efficiency could be observed by diluting the samples (considering samples at 20% and 40% ABV). Neither the sensory dataset for one or the other dilution was sufficient to train an LDA with a similar efficiency as the combined dataset. While other models such as ANN might be able to succeed with either concentration, LDA was a suitable model as it yields influence values (the delta predictor) that explain what factors were decisive in the classification.

#### Differentiation between Scotch and American whiskies

**Fig. 2:**
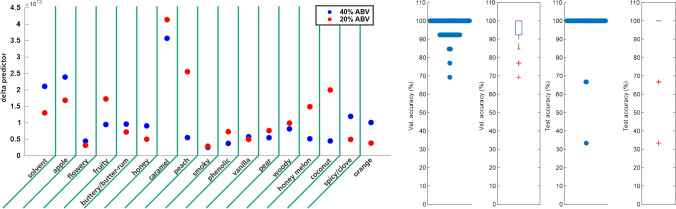
Linear discriminant analysis of the sensory data of American and Scotch whisky samples at 20 % and 40 % ABV (alcohol by volume). Left: relevance of single sensory attributes as predictors for the categorization. The delta predictor indicates the influence of a single attribute on the differentiability between classes. Right: differentiation between Scotch and American whisky. Validation accuracy shows one value for one set of 13 samples (12/13 correctly predicted equals 92.3%, etc.) in 5000 training repetitions with cross-validation. Test accuracy shows the same for 5000 repetitions of testing on a set of 3 samples (e.g., 2/3 correctly predicted equals 66.6% test accuracy)

A linear discriminant analysis (LDA) was performed on the sensory RATA data (mean over participants) to gain further insights into the differences and similarities between the samples. For this purpose, there was one data point for each whisky comprising information for both the 20% and 40% ABV level, as these are no independent samples. Based on this, we were able to categorize the aroma characteristics of the samples in Scotch and American whiskies with high accuracy. Within 5000 repetitions of training with randomly drawn sets of 13 and testing with the remaining 3 samples, 97.86% of classifications in the test set were correct (Fig. 2, right side; avg. testing accuracy 99.03%, avg. validation accuracy 96.93%). As further illustrated in Fig. 2 (left side), it could be shown that the attribute caramel/cream caramel/toffee-like could thereby be used as a reliable predictor for the differentiation. The determination of predictive attributes for the differentiation between Scotch and American whiskies was also achieved by Ashmore et al. [35]. The authors identified some smoke-related attributes (“rubber,” “bacon,” and “peat-smoke”) as possible predictors for peated single malt Scotch as well as the attributes “oak” and “vanilla” for American whiskies.

### Analysis of model whiskies

**Fig. 3:**
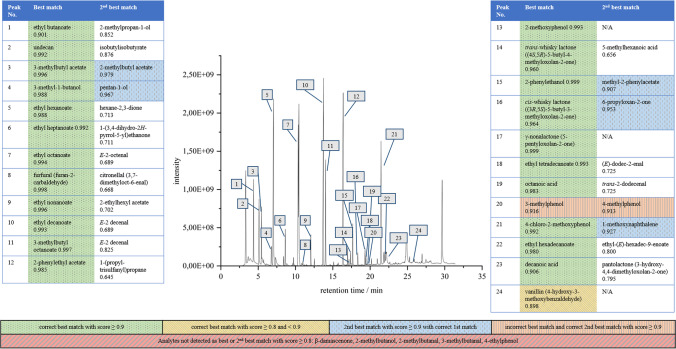
Correctly assigned analytes in model whisky 1. All analytes designated as “best match” present in model whisky 1 as well as one analyte suggested as 2^nd^ best match are shown together with the respective 2^nd^ best matches, chromatogram was considered before blank subtraction. If analytes were detected at several retention times, matches with highest peak areas were considered. Analytes present in the model whisky that have not been detected, are indicated at the bottom

To evaluate the extraction efficiency of the applied sampling method as well as to test the reliability of the semi-automated processing, two model whiskies were analyzed. The persons handling the automated processing program for the analytical data were not informed about the compositions of the model whiskies at first to avoid any influencing factors. The models contained 27 (model whisky 1) and 26 (model whisky 2) typical whisky aroma compounds in varying concentrations (cf. Table 1). Applying a threshold of 0.8 for the total score (Eq. 2) and a ± 30 penalty for the L-RI value (Eq. 3) for the detection of analytes, 21 out of 27 compounds (model whisky 1) and 21 out of 26 compounds (model whisky 2) were eventually detected correctly after blank subtraction (as described above). The internal standards 4-chloro-2-methoxyphenol and undecane were detected in both model whiskies with scores > 0.9. In model whisky 1, all added analytes were detected except for *β*-damascenone, 2-methylbutanol, 4-methylphenol, 4-ethylphenol and the two aldehydes 2- and 3-methylbutanal. An overview on the detection of analytes in model whisky 1 is shown in Fig. 3. Instead of 4-methylphenol and 4-ethylphenol, the respective C3-isomers 3-methylphenol and 3-ethylphenol were reported as best matches. This might be attributed to the fact that the isomers show similar RI values and mass spectra. Consideration of the second-best hit showed that 4-methylphenol was listed right after 3-methylphenol with a slightly lower score (0.913 and 0.916). 2-Methylbutanol was not reported using the automated processing, while 3-methylbutanol with similar RI-value and mass spectrum was detected at multiple retention times. The second-best match, however, was not 2-methylbutanol. *β*-Damascenone was not detected by the automated processing which might be because of low analyte concentrations or extraction efficiency during the SBSE. It can be assumed that the assessment and detection of these two analytes is impeded, especially at low concentrations. Processing of chromatographic data of model whisky 2 led to the detection of 21 out of 26 analytes added to the model whisky; 2-methoxyphenol, furfural, 2- and 3-methylbutanal, and phenol were not reported. The former is a phenolic compound with a polar hydroxyl- and methoxy group showing a log K_O/W_ value of around 1.3 and boiling point of 205 °C [43]. Relatively high polarity as represented by a low log K_O/W_ might result in poor extraction by the unpolar PDMS sorbent. As the compound was detected in model whisky 1, however, it could be shown that the applied method is suitable for higher analyte concentrations (0.54 µg/mL vs 3.36 µg/mL). Other phenolic compounds like 4-allyl-2-methoxyphenol and 4-methylphenol were assigned correctly in model whisky 2. Phenol and furfural were only detected in the original model whisky 2 data before blank subtraction. As both analytes also occurred in the blank chromatograms in similar intensities, they were subtracted from the model whisky results. The detection of these analytes in the blank analyses might be due to contaminations, e.g., in the chromatographical system as well as the high sensitivity of the applied compound detection procedure. It should be noted that, apart from the compounds that were intentionally added to the model whiskies, many other compounds were detected applying the automated processing on the chromatographic data. This means that there is generally a high background noise that is sensitively detected by the automated processing.

Taken together, the majority of analytes were successfully extracted and detected in the model whiskies. However, for a few compounds, the applied analytical methods and subsequent automated data processing is not yet capable to provide a reliable detection and compound assignment. This is, for example, the case for 2-methoxyphenol and *β*-damascenone, which might only be detectable at higher concentrations. For other analytes, a clear distinction between isomers could not be achieved. Therefore, it can be assumed that in some cases, the analytical procedure (e.g. extraction efficiency) can be regarded as the limiting factor, while for other compounds, the automated processing needs further tuning. This might be achieved through less sensitive peak detection algorithms to exclude noise and the training of models (e.g., decision trees) for the correct identification of isomers based on differences in mass spectral data. For this, it is likely that few m/z-peak ratios specific to an isomer would be sufficient. Furthermore, the results show that a score of 0.8 and RI value penalty of ± 30 is appropriate for the analyte detection in the present samples.

### Detection of volatile compounds in whisky samples

Applying a score of 0.8 and a RI value penalty of ± 30 a chromatographic dataset obtained from 16 different whiskies was processed in an automated compound detection. As described above, this includes a semi-automated process for compound detection and identity proposition based on MS and RI data (Eqs. 1 and 2) without the consideration of odor activities or qualities, the direct comparison with reference measurements, or further evaluation by experts. The results discussed here refer to the analytes assigned as best matches following the semi-automated compound detection based on RI and MS characteristics in comparison to an in-house built analyte database. In all whisky samples, the two added internal standards undecane and 4-chloro-2-methoxyphenol were successfully detected. Between 89 (Scotch sample S07B400) and 153 (Scotch Sample S04A400) analytes were proposed in the whiskies. In total, 279 compounds were reported for the 16 samples (cf. Tables S5 and S6), including internal standards. It is important to note that all listed compounds have been solely detected by the semi-automated evaluation and that no further methods of unequivocal compound identification, e.g., comparison with reference standards, have been used. Some resulting substances are uncommon for whiskies. This might become obvious especially for some compounds that also occur in any blank chromatograms (for model whiskies or samples as described above). Compounds also found in blank chromatograms, with comparably low confidence values (< 0.9) or other not reported as typical whisky compounds, are therefore marked (cf. Tables S5 and S6) to avoid misinterpretations. Nevertheless, among these, there were many compounds that have previously been reported for whisky [5, 6, 8, 9]. Among the compounds that were reported in all 16 samples (cf. Table S4) following the described compound detection procedure were decanoic acid, ethyl decanoate, 3-methylbutanol, dodecanoic acid, ethyl octanoate, (*Z*)-hexadec-9-enoic acid, octanoic acid, 2-phenylethyl acetate, ethyl dodecanoate, and ethyl hexanoate. These analytes belong to those showing the highest relative areas in relation to 4-chloro-2-methoxyphenol; however, this is no indication of their concentration ratios or influence on the overall whisky aroma. Typical whisky volatiles [4–6, 9] found in the samples were, for example, vanillin, *cis/trans* whisky lactone, 2-methoxyphenol, 2-phenylethanol, 4-allyl-2-methoxyphenol, and further ethyl and isoamyl esters. Vanillin was proposed for all American whisky samples, and 4 Scotch, *cis* or *trans* whisky lactone was found in all 16 samples, while 4-allyl-2-methoxyphenol (eugenol) was detected in all American and 3 Scotch whiskies. Surprisingly, 2-methoxyphenol (guaiacol) was detected in all American and only 5 Scotch whiskies. 2-Phenylethanol was detected in all 16 samples. Apart from the already mentioned ethyl esters, further examples of this compound class are ethyl heptanoate, ethyl butanoate, and ethyl nonanoate. Other esters proposed for some samples include isopentyl decanoate, isoamyl hexanoate, isoamyl octanoate, and isoamyl acetate. Some analytes that were not detected in the model whiskies (before or after blank subtraction) were however reported for at least one whisky sample. These include 2-methylbutanol, *β*-damascenone, and 3-methylbutanal. Several phenolic compounds, such as 4-ethyl-2-methoxyphenol, 3- or 4-methylphenol, and 3- or 4-ethylphenol, were also proposed for some samples. Apart from whisky lactone, other representatives of the group of lactones, as for example, γ-decalactone and γ-nonalactone, were reported. Apart from a wide spectrum of analytes regarding functional groups and physico-chemical properties, a variety of different odor qualities was represented by the detected analytes, characteristic for whisky [1, 4, 5]. This for example includes compounds with a fruity (ethyl hexanoate, isoamyl acetate), flowery (2-phenylethanol, 2-phenylethyl acetate), phenolic/smoky or burnt (2-methoxyphenol, 4-methylphenol, 4-ethylphenol), or coconut-like (γ-nonalactone, whisky lactones) aroma perception (aroma qualities obtained from in-house database and [4, 5]).

#### Classification of Scotch and American whisky based on analytical results

**Fig. 4:**
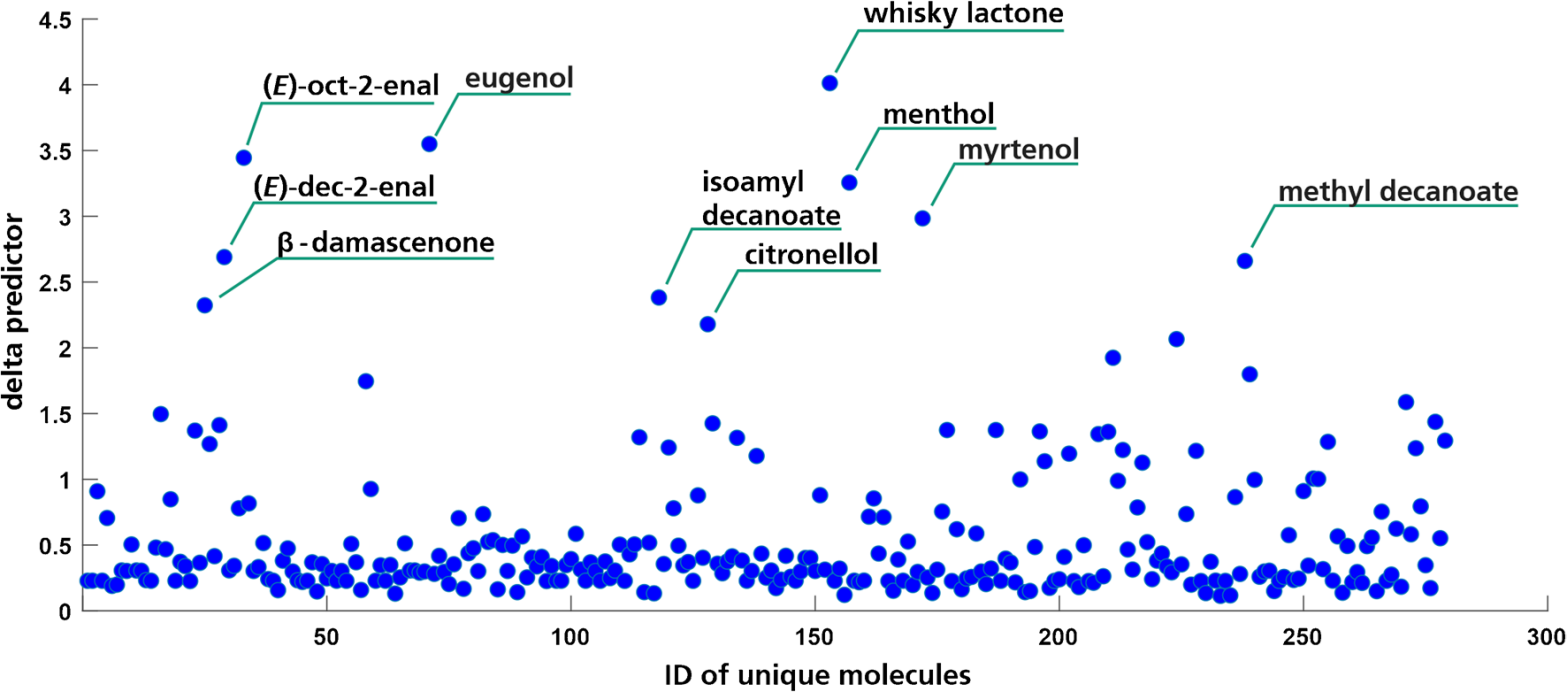
Determination of potential predictors for the classification of Scotch and American whisky based on the delta predictors of an LDA using all detected whisky compounds. A total of 10 potential predictors were determined: (*E*)-2-octenal, (*E*)-dec-2-enal, eugenol (2-methoxy-4-prop-2-enylphenol), whisky lactone (5-butyl-3-methyloxolan-2-one), *β*-damascenone ((*E*)-1-(2,6,6-trimethylcyclohexa-1,3-dien-1-yl)but-2-en-1-one), 3-methylbutyl decanoate, citronellol (3,7-dimethyloct-6-en-1-ol), menthol (5-methyl-2-propan-2-ylcyclohexan-1-ol), myrtenol ((6,6-dimethyl-2-bicyclo[3.1.1]hept-2-enyl)methanol), and methyl decanoate

We found that several molecules could be used in a binary decision process to distinguish between the two whisky types. Inspection of the data showed that two molecules were only proposed for Scotch and for all Scotch samples, namely heptanoic acid and methyl decanoate, while two other compounds, 3,7-dimethyloct-6-en-1-ol (citronellol) and 5-methyl-2-propan-2-yl-cyclohexan-1-ol (menthol), were detected exclusively in all American whiskies. Despite finding the molecules menthol (exclusive to American whisky) and heptanoic acid (exclusive to Scotch) in the blank chromatogram, the peak areas were consistently far lower than in sample chromatograms. Menthol peak area in the blank chromatogram was 5.9% of the average peak area in all American whisky samples, while heptanoic acid peak area equalled 11% of the average in all Scotch samples. We therefore still propose these molecules to be valuable in the classification of the whisky types. This data-driven determination of exclusive molecules that can be used for distinction is promising, while its validity still needs to be evaluated more rigorously. As only a small number of samples (9 Scotch, 7 American Whiskies) was analyzed, the use of these compounds as distinct predictors for the two whisky types has to be further investigated. According to literature findings, methyl decanoate has so far been detected in Scotch whisky [2, 7, 9]; no report on methyl decanoate in American whisky has been found to the best of our knowledge. González-Arjona et al. [18] investigated three different whisky types (Scotch, Irish, and Bourbon) via GC–MS analysis of the solvent extracts and evaluated different pattern recognition procedures for their classification. After conducting a PCA on their data, they identified heptanoic acid among others as one of the features contributing the most to the first two PCs. However, they did not indicate in which samples the compound was predominantly found [18]. While heptanoic acid and methyl decanoate have been reported for whisky [2, 7, 9], citronellol and menthol do obviously not represent typical whisky components. Interestingly, three of the four molecules (except heptanoic acid) were also among the most decisive factors in the LDA-mediated classification (see Figs. 4 and 6). Apart from that, it could be seen that among the compounds that were only detected in Scotch samples, but not necessarily in all of them, were several phenolic compounds. This is in accordance with previous reports, where especially for peated whisky, different phenolic compounds were found [4, 44].

Furthermore, an LDA was conducted based on the analytes detected in the 16 whisky samples to see if the two kinds can be classified based on their automatically generated compounds lists as described above. For this purpose, all unique analytes (analytes could only appear once in each sample) were considered in a semi-quantitative manner by calculating the relative peak area to the internal standard 4-chloro-2-methoxyphenol. Figure 4 shows the main influences for the classification as determined by LDA. Ten molecules with delta predictor values above 2 are labeled as they showed the highest influence among all molecules. These were (*E*)-2-octenal, (*E*)-dec-2-enal, eugenol (2-methoxy-4-prop-2-enylphenol), whisky lactone (5-butyl-3-methyloxolan-2-one), *β*-damascenone ((*E*)-1-(2,6,6-trimethylcyclohexa-1,3-dien-1-yl)but-2-en-1-one), 3-methylbutyl decanoate, citronellol (3,7-dimethyloct-6-en-1-ol), menthol (5-methyl-2-propan-2-ylcyclohexan-1-ol), myrtenol ((6,6-dimethyl-2-bicyclo[3.1.1]hept-2-enyl)methanol), and methyl decanoate. While some of these compounds like eugenol, whisky lactone, isoamyl decanoate, *β*-damascenone, and methyl decanoate have been reported for different kinds of whisky [2, 5–7, 9], the other compounds do not represent typical whisky volatiles as shown by literature (cf. Table S4–6). However, as noted above, none of those compounds have been identified unequivocally. With this approach, potential marker compounds for a targeted analysis have been revealed that might be useful for conducting a classification solely based on selected compounds. For a validated targeted analytical method, the identification of all compounds using reference standards, as well as a control experiment by olfactometric evaluation, should be added. This method development, however, is strongly facilitated by the two factor determined (L-RI and MS matching) list of potential targets. For sample classification on the other hand, the consideration of all analytes detected, as conducted within the present research, tolerates the appearance of additional compounds and might also refine the classification in some cases as more variables are included.

The delta predictor plotted in Fig. 4 is a representation of how much of an influence each individual molecule exerted on the classification. The compound with the highest value is whisky lactone (sum of *trans* and *cis* whisky lactone), which was detected in all whisky samples. However, the relative peak areas (compared to the internal standard 4-chloro-2-methoxyphenol) indicated that the analyte was present in higher amounts in the American whiskies.

**Fig. 5:**
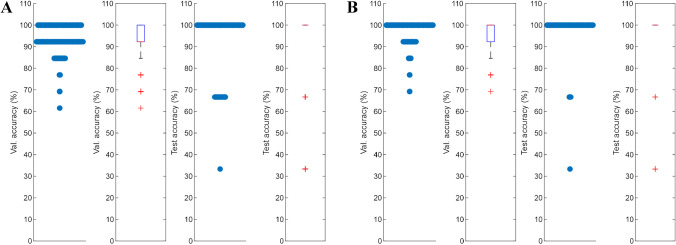
Linear discriminant analysis for the classification of 16 whisky samples based on analytical data, 5000 repetitions using a training set of 13 and test set of 3 samples. **A** without and **B** with preceding PCA keeping only 4 main components, leading to accuracies of 80.74% and 96.98%, respectively

LDA on analytical data performed by the procedure outlined above resulted in 80.74% prediction accuracy for the classification between Scotch and Bourbon based on the detected volatiles (Fig. 5A). This relatively low value might be ascribed to several causes: In the whisky samples, a significant number of minor compounds were detected, and there was no subtraction of a blank chromatogram. Therefore, many compounds that are not relevant for the classification of whiskies or occur only in traces or as noise are also considered here. This might be a reason for the occurrence of rather untypical whisky volatiles as predictors as well as low prediction accuracy. A high number of predictors in combination with a low sample count further pose the risk of overfitting in LDA. This concern was addressed by introducing a preceding PCA keeping only 4 main components, which led to an accuracy of 96.98% (Fig. 5B). To avoid this issue, further thresholds could be set, e.g., in relation to minimum peak area or compound purity to improve the processing of GC–MS data. Furthermore, low classification accuracy might be attributed to the quality of the generated GC–MS data. As compounds of various different compound classes and physico-chemical features appear in a wide concentration range, their sampling and analysis must be suitable to detect differences between the samples. As analyte extraction was conducted using a PDMS Twister®, extraction efficiency for polar compounds present in lower concentrations might have been insufficient. Furthermore, it could be seen that especially ethyl esters such as ethyl decanoate and ethyl dodecanoate were extracted in high amounts from all samples, irrespective of the type of whisky. Taken together, the samples showed huge similarities especially for the major peaks detected in the chromatograms. For more detailed information on analytical differences between the samples, other sampling strategies, e.g., the use of other sorbent phases, should be considered. Furthermore, the use of specifically designed databases for whisky samples as opposed to general in-house (aroma) compound database that was used for this study could refine the analyte list. This would put a stronger focus on analytes relevant for whisky, as suggested by Grasskamp et al. [39].

A third approach showed a classification accuracy of 94.2% with analytical data, but with all molecule amounts turned to 1 (resulting in a binary, qualitative analytical matrix), without the consideration of (relative) peak areas. The results of this are shown in the supplementary material (online resources Fig. S1). This analysis put a heavy focus on those molecules that were only detected in one of the two whisky types. As whisky lactone showed a clear and systematic distinction in detected amounts between the two whisky types, but was found in each sample, it had no influence in this type of qualitative analysis. Furthermore, many of the molecules determined as having the highest influence on the separation were those detected with low confidence values close to the cut-off of 0.8. This may point towards closely related compounds of whisky specific molecules that were not present in our target database or towards problems in purity of trace compounds essential for the distinction.

### Comparison of the use of sensory and analytical data for whisky classification

**Fig. 6:**
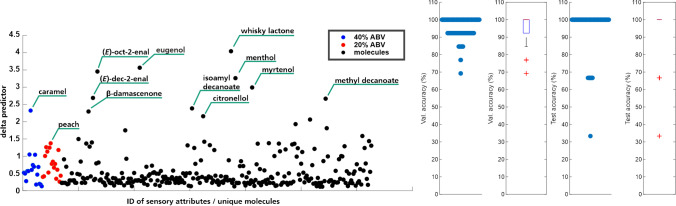
Linear discriminant analysis for the classification of 16 whisky samples based on analytical and sensory data in combination, 5000 repetitions using a training set of 13 and test set of 3 samples. Indication of potential predictors based on chemical analysis ((*E*)-2-octenal, (*E*)-dec-2-enal, eugenol (2-methoxy-4-prop-2-enylphenol), whisky lactone (5-butyl-3-methyloxolan-2-one), *β*-damascenone ((*E*)-1-(2,6,6-trimethylcyclohexa-1,3-dien-1-yl)but-2-en-1-one), 3-methylbutyl decanoate, citronellol (3,7-dimethyloct-6-en-1-ol), menthol (5-methyl-2-propan-2-ylcyclohexan-1-ol), myrtenol ((6,6-dimethyl-2-bicyclo[3.1.1]hept-2-enyl)methanol) and methyl decanoate), and sensory evaluation (caramel, peach)

Eventually, a LDA was conducted using both the analytical and sensory data in combination. In agreement with both the LDA from sensory as well as from analytical data, the classification accuracy of the combinatory LDA was found to be between the two single LDAs. Within 5000 repetitions of training with 13 and testing with 3 samples, correct classification was achieved in 87.96% (Fig. 6).

While significantly more predictors showed an influence above 2.0 (delta predictor) for the analytical data, the sensory attribute “caramel” showed by far the highest value among all attributes for the sensory data. Even so, the classification of whisky types applying LDA was more accurate using sensory data (97.86%) than semi-quantitive analytical data without preceding PCA (80.74%). From this, it can be concluded that the sensory attributes used are more distinctive for the two kinds of whiskies than the compounds detected using the semi-automated processing following SBSE-GC–MS analysis. As discussed above, this might be due to the large number of analytes including minor compounds and noise detected in the whisky samples. Interestingly, while the most distinctive sensory attributes according to the LDA were caramel-like and peach-like, the odor qualities of the descriptor analytes range from fatty ((*E*)-oct-2-enal, (*E*) 2-dec-2-enal), grape-juice/cooked apple like (*β*-damascenone), eucalyptus-like (menthol, myrtenol), soapy (methyl decanaote), clove-like (eugenol) to lemongrass-like, rose-like (citronellol), and coconut-like (whisky lactone) (aroma qualities obtained from in-house database and [5]). Therefore, for an automated evaluation on how certain molecules might define or influence the sensory characteristics of a sample, other features might have to be taken into account. For odorous compounds, it has to be considered that their concentration within the sample does not necessarily correlate with their impact on the overall aroma as they might show high odor thresholds and therefore lower odor activities as compared to other compounds. Thus, even compounds present in low concentrations might have a significant influence on the aroma characteristics of the whisky. Within comprehensive aroma or odorant analyses, e.g., for the determination of key aroma or odor compounds, often a correlation between the most potent odorants (as characterized by high flavor or odor dilution factors) and the predominant sensory attributes can be observed [11, 45, 46]. For this purpose, odor qualities as well as odor thresholds and resulting odor activity values [11, 28, 47] for the detected compounds could be included in the presented automated processing. This would not only allow for a potential characterization of the sensory properties but also combine analytical and sensory classifications of the samples based on solely analytical data.

Based on the two datasets described here, the use of sensory data and analytical data are both suited for the classification of Scotch and American whisky. However, even if a fast sensory method (RATA) was applied, sensory evaluation is a time-consuming step, particularly for large sample sets. Especially for classification matters, more efficient methods based on analytical data are more appropriate. Therefore, analytical data generated by high-throughput GC–MS treated by semi-automated processing might soon provide the most efficient method for this objective.

## Summary and conclusions

Within this paper, the analytical as well as sensory evaluation of 16 different whisky samples was presented. Focus thereby laid on the evaluation of efficient analysis methods and data processing for both chemical and sensory evaluation. Statistical methods were applied to gain deeper insights into the generated data and to compare the two approaches for a classification problem (Scotch vs. American whisky). The present article shows how simplified analytical data and rapid sensory evaluation can be successfully used for an automated classification of whiskies into Scotch and Bourbon. While the scope of this research was not to thoroughly and unequivocally identify (key) aroma compounds in the samples, it is an example of how automated processes and statistical analysis can assist the handling and evaluation of samples, e.g., to get a qualitative overview on larger datasets based on automatically processed GC–MS data.

Sensory evaluation and subsequent statistical analysis showed that from the 17 attributes offered, the following eight contributed significantly to the overall differentiation of samples: Caramel/cream caramel/toffee-like, vanilla-like, (canned) peach-like and (fermented) apple/cider-like. Performing an LDA, the samples were classified with high accuracy (97.86%) into Scotch and American whisky. The attribute Caramel/cream caramel/toffee-like was thereby identified as a reliable predictor for the differentiation of Scotch and American whisky in the present sample set.

The analysis of two model whiskies including 26 and 27 typical whisky compounds, respectively, suggests that the analytical procedure, including sample analysis as well as processing, is suitable for the detection of aroma compounds in whisky samples with some drawbacks. Applying the same analytical procedure to a set of 16 whiskies (American and Scotch), a large number of (aroma-active) volatile compounds, including compounds typically found in whisky, were detected. An LDA was then conducted on the data to evaluate if the samples can be classified into Scotch and American whisky based on the analyte lists. Results show an accuracy of 80.74% for the semi-quantitative data (including relative peak areas) and 94.2% with qualitative data only, while a preceding PCA keeping four main components (explaining > 95% of observed variance) increases these values to 96.94% and 99.92%, respectively. Compared to sensory data, the use of the analytical data of the proposed classification problem offers similar accuracy.

Apart from the optimization and adjustment of analytical parameters, the development of semi-automated and in-house fitted data processing platforms is a crucial aspect. Next to the processing of chromatographical data, this also includes advanced strategies for analyte identification, e.g., based on MS data in comparison to reference compounds. The use of modern data science and machine learning has become an integral part in analysis of MS with different modes of chromatography (liquid/gas), showing how an approach such as ours may well be amended to further improve outcomes. Several as yet unsolved issues can be addressed with approaches such as this. Among these, taking into account what margin of error may be expected from different detectors is an inherently fitting problem for highly performant data analysis methods and will not be solved by manual work due to the complexity. This necessitates a systematic large-scale investigation of mass spectra aided by efficient approaches with high potential to aid in human decision-making such as the one presented here.

Taken together, our investigations show the potential of efficient sensory and analytical procedures for the evaluation of whisky samples based on their aroma characteristics and volatiles profile. A simple sample workup and analysis procedure (SBSE GC–MS) including the semi-automated analyte detection and proposition as well as a rapid sensory method (RATA) were tested and evaluated in combination with statistical methods for their potential to solve a simple classification problem. This represents a step towards a comprehensive platform for fast and efficient aroma analysis. Thereby, more sophisticated classification questions, e.g., in relation to aroma characteristics of food samples based on analytical data, can be approached. For this purpose, further investigations can be conducted to adjust and test analytical parameters for analyte extraction and instrumental analysis as well as the data processing, analyte identification, and the consideration of compound specific properties, such as odor activity values. The prediction of whisky aroma features based on analytical data using chemometrics or even machine learning models would be beyond the scope of this paper. However, the present article presents some first and promising steps for the implementation of a fast and efficient platform for the generation and handling of analytical and sensory data for aroma analysis.

### Supplementary Information

Below is the link to the electronic supplementary material.

Supplementary file1 (DOCX 259 KB)

## Data Availability

Data generated during the current study are available from the corresponding authors on reasonable request.
