# Supplementary material for: Quick insights into whisky — investigating rapid and efficient methods for sensory evaluation and chemical analysis
Source: Anal Bioanal Chem. 2023 Sep 2;415(24):6091–106. doi: 10.1007/s00216-023-04883-5 (PMC10556146; doi:10.1007/s00216-023-04883-5)
Supplement: Supplementary file 1 — Supplementary file1 (DOCX 259 KB) [file 216_2023_4883_MOESM1_ESM.docx]

**Quick insights into whisky – investigating rapid and efficient methods for sensory evaluation and chemical analysis**

Helen Haug^1, 2^, Andreas T. Grasskamp^1^, Satnam Singh^1^, Andrea Strube^1^ and Tilman Sauerwald^1^

^1^ Department of Sensory Analytics & Technologies, Fraunhofer Institute for Process Engineering and Packaging IVV, Giggenhauser Str. 35, 85354 Freising, Germany

^2^ Chair of Aroma and Smell Research, Department of Chemistry and Pharmacy, Friedrich-Alexander-Universität Erlangen-Nürnberg, Henkestraße 9, 91054 Erlangen, Germany

Helen Haug and Andreas T. Grasskamp contributed equally to the work and are shared first authors.
Corresponding author email: Helen Haug [helen.haug@ivv.fraunhofer.de](mailto:helen.haug@ivv.fraunhofer.de); Tilman Sauerwald tilman.sauerwald@ivv.fraunhofer.de

**Supplementary information (SI)**

**Table S1** Chemicals (grade and supplier)

| Compound | Supplier and grade |
| --- | --- |
| *β*-Damascenone ((*E*)-1-(2,6,6-trimethylcyclohexa-1,3-dien-1-yl)but-2-en-1-one) | Sigma Aldrich, Steinheim, Germany; 1.1 – 1.4 % wt. |
| 2-Phenylethanol | Sigma Aldrich, Schnellendorf, Germany; 99+ % |
| 3-Methylbutyl acetate | Sigma Aldrich (Fluka), Steinheim, Germany; anal. Std. |
| Phenylethyl acetate | Sigma Aldrich (Fluka), Steinheim, Germany; ≥ 99 % |
| 3-Methylbutyl octanoate | Sigma Aldrich, Steinheim, Germany; ≥ 98 % |
| 3-Methylbutyl decanoate | Sigma Aldrich, Steinheim, Germany |
| Ethyl hexanoate | Sigma Aldrich, Steinheim, Germany; ≥ 99 % |
| Ethyl heptanoate | Sigma Aldrich, Steinheim, Germany; ≥ 98 % |
| Ethyl octanoate | Sigma Aldrich, Steinheim, Germany; ≥ 98 % |
| Ethyl decanoate | Sigma Aldrich, Steinheim, Germany; 99+ % |
| Ethyl nonanoate | Sigma Aldrich, Steinheim, Germany; ≥ 98 % |
| Ethyl tetradecanoate | Sigma Aldrich, Steinheim, Germany; ≥ 98 % |
| Ethyl hexadecanoate | Thermo Fisher (Alfa Aesar), Kandel, Germany; 0.97 % |
| Octanoic acid | Sigma Aldrich, Steinheim, Germany; 98+ % |
| Decanoic acid | Sigma Aldrich, Steinheim, Germany; 98+ % |
| Dodecanoic acid | Sigma Aldrich, Steinheim, Germany; 98 % |
| Furfural (furan-2-carbaldehyde) | Sigma Aldrich, Steinheim, Germany; ≥ 98 % |
| 2-Methoxyphenol | Sigma Aldrich, Steinheim, Germany; ≥98 % |
| 3-Methylbutanal | Sigma Aldrich, Steinheim, Germany; 97 % |
| 2-Methylbutanal | Sigma Aldrich, Steinheim, Germany; 95 % |
| 2-Methyl-1-butanol | Sigma Aldrich, Steinheim, Germany; 99+ % |
| Ethyl butanoate | Sigma Aldrich (Fluka), Steinheim, Germany; ≥ 98% |
| Phenol | Sigma Aldrich (Fluka), Steinheim, Germany; > 99% |
| 4-Methylphenol | Sigma Aldrich, Steinheim, Germany; 99 % |
| 4-Ethylphenol | Honeywell (Riedel de Haen), Seelze, Germany; 99 % |
| Whisky-lactone cis/trans (5-butyl-3-methyloxolan-2-one) | Sigma Aldrich, Steinheim, Germany; 98+ % |
| γ-Nonalactone (5-pentyloxolan-2-one) | Sigma Aldrich, Steinheim, Germany; ≥ 98% |
| 4-Allyl-2-methoxyphenol (2-methoxy-4-prop-2-enylphenol) | Sigma Aldrich, Steinheim, Germany; 99% |
| 4-Hydroxy-3-methoxy-benzaldehyde | ABCR, Karlsruhe, Germany; 99% |
| 3-Methylbutanol | Sigma Aldrich (Fluka), Steinheim, Germany; 99 % |
| *n*-Undecane | Sigma Aldrich, Steinheim, Germany; 99+ % |
| 4-Chloro-2-methoxyphenol | Sigma Aldrich, Steinheim, Germany; techn. grade |
| Sodium chloride | Th. Geyer GmbH & Co. KG, Renningen, Germany; 99% |
| Ethanol | VWR International GmbH, Darmstadt, Germany; absolute |

Whisky samples

**Table S2** Whisky samples for sensory and chemical analysis.

| Name | Type | Origin | Alcohol Content |
| --- | --- | --- | --- |
| Auchentoshan 12 Years old | Scotch, Single Malt | Scotland, Lowlands | 40 % vol. |
| Bowmore 12 Years old | Scotch, Single Malt | Scotland, Islay | 40 % vol. |
| Talisker Isle of Skye Malt - 10 Years | Scotch, Single Malt | Scotland, Isle of Skye | 45.8 % vol. |
| Laphroaig 10 Years old | Scotch, Single Malt | Scotland, Islay | 40 % vol. |
| Glenfarclas 10 Years Old | Scotch, Single Malt | Scotland, Speyside | 40 % vol. |
| Glengoyne 10 Years Highland | Scotch, Single Malt | Scotland, Highlands | 40 % vol. |
| Glenkinchie 12 Years old | Scotch, Single Malt | Scotland, Lowlands | 43 % vol. |
| Glenmorangie Original | Scotch, Single Malt | Scotland, Highlands | 40 % vol. |
| Johnnie Walker Red Label - Old Scotch Whisky | Scotch, Blend | Scotland, Highlands | 40 % vol. |
| Bulleit Rye Frontier Whiskey | Straight Rye | Kentucky, USA | 45 % vol. |
| Four Roses Single Barrel | Kentucky Straight Bourbon | Kentucky, USA | 50 % vol. |
| Jack Daniels - Tennessee Whiskey | Tennessee Whisky | Tennessee, USA | 40 % vol. |
| Maker's Mark Whisky | Kentucky Straight Bourbon | Kentucky, USA | 45 % vol. |
| Wild Turkey 101 Proof | Kentucky Straight Bourbon | Kentucky, USA | 50.5 % vol. |
| Woodford Reserve Bourbon | Kentucky Straight Bourbon | Kentucky, USA | 43.2 % vol. |
| Knob Creek | Kentucky Straight Bourbon | Kentucky, USA | 50 % vol. |

**Table S3**: Whisky samples and identifiers for sensory and chemical analysis.

| Whisky | Alcohol content  (original, o /  adjusted, a) | Identifier |
| --- | --- | --- |
| Auchentoshan 12 Years old | 40 % vol. (o) | S01A400 |
| Bowmore 12 Years old | 40 % vol. (o) | S02A400 |
| Talisker Isle of Skye Malt - 10 Years | 40 % vol. (a) | S03B400 |
| Laphroaig 10 Years old | 40 % vol. (o) | S04A400 |
| Glenfarclas 10 Years Old | 40 % vol. (o) | S05A400 |
| Glengoyne 10 Years Highland | 40 % vol. (o) | S06A400 |
| Glenkinchie 12 Years old | 40 % vol. (a) | S07B400 |
| Glenmorangie Original | 40 % vol. (o) | S08A400 |
| Johnnie Walker Red Label - Old Scotch Whisky | 40 % vol. (o) | S09A400 |
| Bulleit Rye Frontier Whiskey | 40 % vol. (a) | B10B400 |
| Four Roses Single Barrel | 40 % vol. (a) | B11B400 |
| Jack Daniels - Tennessee Whiskey | 40 % vol. (o) | B12A400 |
| Maker's Mark Whisky | 40 % vol. (a) | B13B400 |
| Wild Turkey 101 Proof | 40 % vol. (a) | B14B400 |
| Woodford Reserve Bourbon | 40 % vol. (a) | B15B400 |
| Knob Creek | 40 % vol. (a) | B16B400 |
| Auchentoshan 12 Years old | 20 % vol. (a) | S01B200 |
| Bowmore 12 Years old | 20 % vol. (a) | S02B200 |
| Talisker Isle of Skye Malt - 10 Years | 20 % vol. (a) | S03B200 |
| Laphroaig 10 Years old | 20 % vol. (a) | S04B200 |
| Glenfarclas 10 Years Old | 20 % vol. (a) | S05B200 |
| Glengoyne 10 Years Highland | 20 % vol. (a) | S06B200 |
| Glenkinchie 12 Years old | 20 % vol. (a) | S07B200 |
| Glenmorangie Original | 20 % vol. (a) | S08B200 |
| Johnnie Walker Red Label - Old Scotch Whisky | 20 % vol. (a) | S09B200 |
| Bulleit Rye Frontier Whiskey | 20 % vol. (a) | B10B200 |
| Four Roses Single Barrel | 20 % vol. (a) | B11B200 |
| Jack Daniels - Tennessee Whiskey | 20 % vol. (a) | B12B200 |
| Maker's Mark Whisky | 20 % vol. (a) | B13B200 |
| Wild Turkey 101 Proof | 20 % vol. (a) | B14B200 |
| Woodford Reserve Bourbon | 20 % vol. (a) | B15B200 |
| Knob Creek | 20 % vol. (a) | B16B200 |


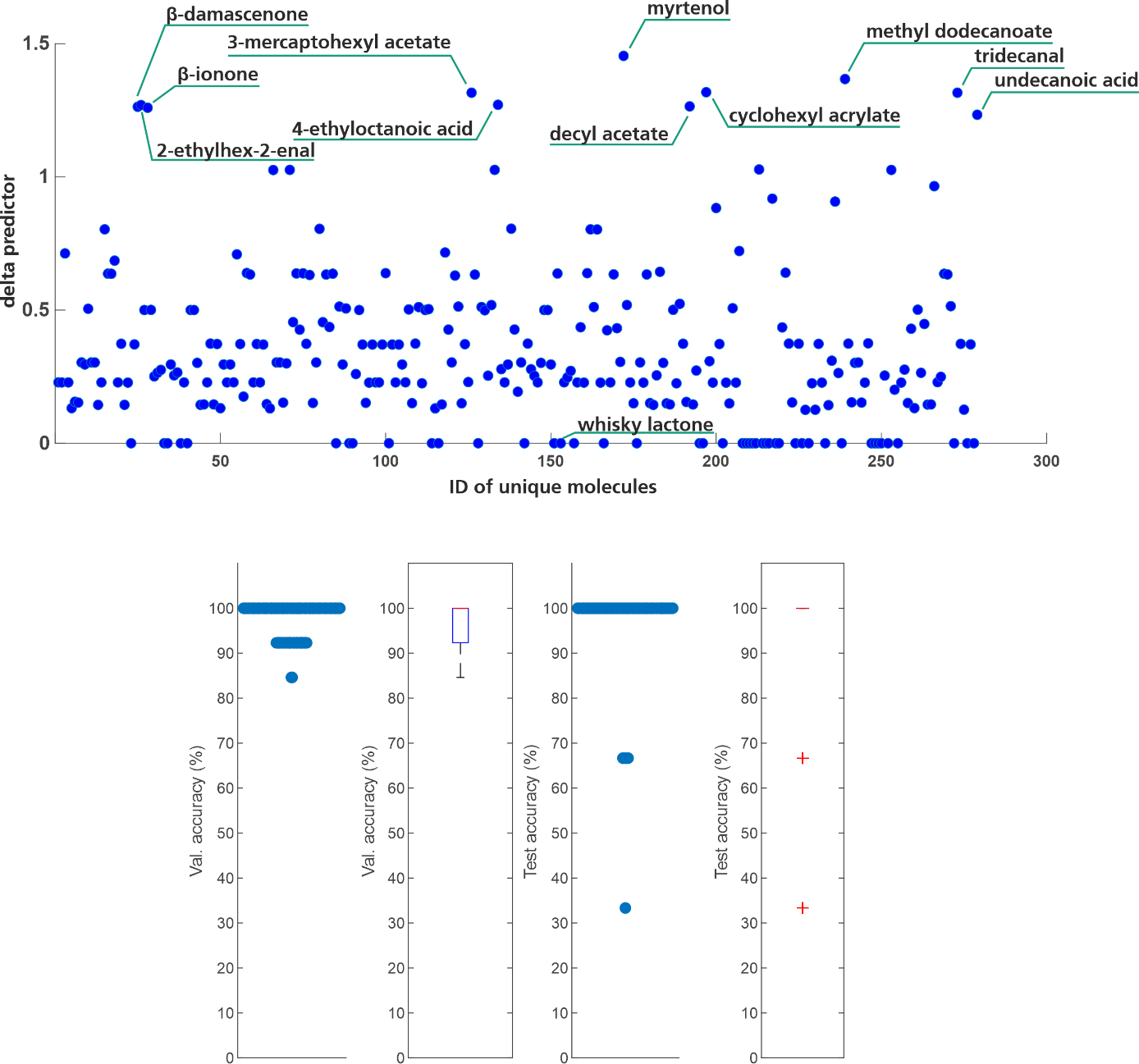


**Fig. S1** Linear discriminant analysis on qualitative analytical data of 16 whisky samples. Indication of 10 potential predictors obtained from qualitative analytical data: β-damascenone ((*E*)-1-(2,6,6-trimethylcyclohexa-1,3-dien-1-yl)but-2-en-1-one), β-ionone ((*E*)-4-(2,6,6-trimethylcyclohexen-1-yl)but-3-en-2-one), 3-mercaptohexyl acetate (3-sulfanylhexyl acetate), 2-ethylhex-2-enal ((*E*)-2-ethylhex-2-enal), 4-ethyloctanoic acid, decyl acetate, myrtenol ((6,6-dimethyl-2-bicyclo[3.1.1]hept-2-enyl)methanol), cyclohexyl acrylate (cyclohexyl prop-2-enoate), methyl dodecanoate, tridecanal, undecanoic acid, whisky lactone (5-butyl-3-methyloxolan-2-one) with low delta predictor.

**Table S4** Alphabetical list of 42 molecules detected in **each** of the 16 whisky samples (internal standards in italics) using an automated compound detection tool and in-house database based on retention index and mass spectral data; with mean confidence (mean match scores for compound detection) over all detected instances at highest relative peak intensity.^1^

| **IUPAC name** | **mean conf.** | **IUPAC name** | **mean  conf.** |
| --- | --- | --- | --- |
| (6*E*)-3,7,11-trimethyldodeca-1,6,10-trien-3-ol | 0.939 | ethyl butanoate | 0.918 |
| *† (*E*)-oct-2-enal | 0.892 | ethyl decanoate | 0.908 |
| (*E*)-undec-2-enal | 0.929 | ethyl dodecanoate | 0.991 |
| *† (*Z*)-hexadec-9-enoic acid | 0.809 | ethyl heptanoate | 0.935 |
| *† 1-(3,4-dihydro-2H-pyrrol-5-yl)ethanone | 0.882 | ethyl hexanoate | 0.905 |
| 2-methylpropyl acetate | 0.904 | ethyl nonanoate | 0.928 |
| 2-phenylethanol | 0.951 | ethyl octanoate | 0.921 |
| 2-phenylethyl acetate | 0.993 | ethyl tetradecanoate | 0.929 |
| 2,4-ditert-butylphenol | 0.955 | furan-2-carbaldehyde | 0.979 |
| 3-methylbutan-1-ol | 0.978 | † hexadecan-1-ol | 0.837 |
| 3-methylbutyl acetate | 0.951 | hexan-1-ol | 0.922 |
| *4-chloro-2-methoxyphenol* | 0.954 | hexyl acetate | 0.927 |
| *† 4-methylnonanoic acid | 0.848 | nonan-2-one | 0.951 |
| 5-(hydroxymethyl)furan-2-carbaldehyde | 0.934 | nonanal | 0.945 |
| 5-butyl-4-methyloxolan-2-one | 0.960 | nonanoic acid | 0.933 |
| *† 6-octyloxan-2-one | 0.839 | oct-1-en-3-ol | 0.939 |
| acetic acid | 0.957 | octan-1-ol | 0.939 |
| † decanal | 0.892 | octanoic acid | 0.973 |
| decanoic acid | 0.958 | tetradecanoic acid | 0.966 |
| dodecanoic acid | 0.962 | undecan-2-one | 0.924 |
| ethyl benzoate | 0.994 | *undecane* | 0.956 |

Table content does neither represent unequivocal compound identification nor a complete list of (aroma active) volatile compounds present in the whisky samples, all molecules should be considered as compound propositions and their presence in the samples has not been sufficiently verified. Furthermore, no reliable statements regarding chemical isomers can be made based on the present data. Marked substances are especially unlikely to be good matches since *they have been found with a confidence below 0.9 and were found in blank chromatograms (relating to the model whisky or the sample analysis) and have not been described in literature to occur in whisky to our knowledge; † substances were detected with confidence below 0.9 and detected in blank chromatograms.

**Table S5** Alphabetical list of molecules detected in **any** of the 16 whisky samples (internal standards in italics) using a semi-automated compound detection tool and in-house database based on retention index and mass spectral data after sorting out substances that are especially unlikely to be found in whisky (cf. Table S6). Indication of mean confidence (mean match scores for compound detection) over all detected instances at highest relative peak intensity.^1^

| **IUPAC name** | **mean conf.** | **IUPAC name** | **mean conf.** |
| --- | --- | --- | --- |
| (2*E*)-3,7-dimethylocta-2,6-dienal | 0.838 | 5-(hydroxymethyl)furan-2-carbaldehyde | 0.934 |
| (2*E*,4*E*)-deca-2,4-dienal | 0.870 | 5-[(*Z*)-oct-2-enyl]oxolan-2-one | 0.941 |
| (2*E*,6*E*)-nona-2,6-dienal | 0.913 | 5-butyl-4-methyloxolan-2-one | 0.960 |
| (3*E*,6*E*)-3,7,11-trimethyldodeca-1,3,6,10-tetraene | 0.898 | 5-methyl-2-propan-2-ylphenol | 0.931 |
| (3*S*)-3,7-dimethyloct-6-enal | 0.835 | 5-methylfuran-2-carbaldehyde | 0.909 |
| (4-methylphenyl) benzoate | 0.921 | 5-octyloxolan-2-one | 0.947 |
| (6*E*)-3,7,11-trimethyldodeca-1,6,10-trien-3-ol | 0.939 | 5-pentyloxolan-2-one | 0.990 |
| (6*E*)-7,11-dimethyl-3-methylidenedodeca-1,6,10-triene | 0.907 | 6-heptyloxan-2-one | 0.887 |
| (*E*)-1-(2,6,6-trimethylcyclohexa-1,3-dien-1-yl)but-2-en-1-one | 0.941 | 6-propyloxan-2-one | 0.824 |
| (*E*)-2-ethylhex-2-enal | 0.817 | 8-tricyclo[5.2.1.0^2,6^]dec-3-enyl acetate | 0.823 |
| (*E*)-3-phenylprop-2-enal | 0.825 | [(1*R*,2*R*,4*R*)-1,7,7-trimethyl-2-bicyclo[2.2.1]heptanyl] 2-methylprop-2-enoate | 0.873 |
| (*E*)-4-(2,6,6-trimethylcyclohexen-1-yl)but-3-en-2-one | 0.866 | [(1*R*,5*S*)-6,6-dimethyl-2-bicyclo[3.1.1]hept-2-enyl]methanol | 0.837 |
| (*E*)-undec-2-enal | 0.929 | [(2*E*)-3,7-dimethylocta-2,6-dienyl] acetate | 0.867 |
| (methyltrisulfanyl)methane | 0.946 | [(*E*)-hex-2-enyl] acetate | 0.931 |
| (*Z*)-hex-3-enal | 0.939 | acetic acid | 0.957 |
| 1-(2-methylphenyl)ethanone | 0.984 | benzaldehyde | 0.969 |
| 1-(4-methoxyphenyl)propan-2-one | 0.961 | benzyl 2-hydroxybenzoate | 0.886 |
| 1-(4-methylphenyl)ethanone | 0.920 | benzyl acetate | 0.866 |
| 1-methoxypropan-2-yl acetate | 0.849 | butan-1-ol | 0.879 |
| 1,2-oxazole | 0.843 | butan-2-yl prop-2-enoate | 0.912 |
| 1*H*-indole | 0.904 | butane-2,3-diol | 0.849 |
| 2-(2-methylprop-2-enoyloxy)ethyl 2-methylprop-2-enoate | 0.862 | butyl acetate | 0.912 |
| 2-(4-methylcyclohex-3-en-1-yl)propan-2-ol | 0.877 | cumene | 0.920 |
| 2-ethoxyphenol | 0.938 | cyclohexyl prop-2-enoate | 0.834 |
| 2-ethyl-3-methylpyrazine | 0.903 | decanoic acid | 0.958 |
| 2-ethylhexyl prop-2-enoate | 0.850 | decyl acetate | 0.983 |
| 2-ethylphenol | 0.910 | dodecanoic acid | 0.962 |
| 2-hydroxyethyl 2-methylprop-2-enoate | 0.825 | ethyl 3-methylbutanoate | 0.897 |
| 2-methoxy-4-prop-2-enylphenol | 0.893 | ethyl benzoate | 0.994 |
| 2-methoxy-4-propylphenol | 0.927 | ethyl butanoate | 0.918 |
| 2-methoxy-5-methylphenol | 0.944 | ethyl decanoate | 0.908 |
| 2-methoxynaphthalene | 0.832 | ethyl dodecanoate | 0.991 |
| 2-methoxyphenol | 0.930 | ethyl heptanoate | 0.935 |
| 2-methylbutan-1-ol | 0.889 | ethyl hexadecanoate | 0.926 |
| 2-methylphenol | 0.931 | ethyl hexanoate | 0.905 |
| 2-methylpropan-1-ol | 0.929 | ethyl nonanoate | 0.928 |
| 2-methylpropyl 2-methylpropanoate | 0.817 | ethyl octanoate | 0.921 |
| 2-methylpropyl acetate | 0.904 | ethyl pentanoate | 0.866 |
| 2-phenylethanol | 0.951 | ethyl tetradecanoate | 0.929 |
| 2-phenylethyl acetate | 0.993 | furan-2-carbaldehyde | 0.979 |
| 2-propan-2-ylphenol | 0.877 | heptan-1-ol | 0.874 |
| 2-sulfanylpentan-3-one | 0.907 | heptan-2-one | 0.921 |
| 2,3,5-trimethylphenol | 0.942 | heptanoic acid | 0.948 |
| 2,3,5-trimethylpyrazine | 0.914 | hexan-1-ol | 0.922 |
| 2,4-dimethylphenol | 0.894 | hexanal | 0.904 |
| 2,4-ditert-butylphenol | 0.955 | hexane-2,3-dione | 0.892 |
| 2,5-dimethylphenol | 0.942 | hexanoic acid | 0.923 |
| 2,6-dimethylphenol | 0.846 | hexyl acetate | 0.927 |
| 2,7-dimethylquinoline | 0.825 | methyl decanoate | 0.850 |
| 3-ethylphenol | 0.969 | methyl dodecanoate | 0.926 |
| 3-methylbutan-1-ol | 0.978 | methyl hexadecanoate | 0.952 |
| 3-methylbutanal | 0.819 | methyl octanoate | 0.898 |
| 3-methylbutyl acetate | 0.951 | n-butan-2-ylidenehydroxylamine | 0.813 |
| 3-methylbutyl decanoate | 0.918 | nonan-2-one | 0.951 |
| 3-methylbutyl hexanoate | 0.955 | nonanal | 0.945 |
| 3-methylbutyl octanoate | 0.980 | nonanoic acid | 0.933 |
| 3-methylbutyl propanoate | 0.889 | oct-1-en-3-ol | 0.939 |
| 3-propan-2-ylphenol | 0.917 | octan-1-ol | 0.939 |
| 3,7-dimethyloct-6-en-1-ol | 0.895 | octan-2-one | 0.976 |
| 3,7-dimethylocta-1,6-dien-3-ol | 0.913 | octanoic acid | 0.973 |
| 4-chloro-2-hydroxybenzonitrile | 0.921 | pent-1-en-3-one | 0.860 |
| *4-chloro-2-methoxyphenol* | 0.954 | pentan-1-ol | 0.850 |
| 4-chlorophenol | 0.862 | phenol | 0.923 |
| 4-ethenyl-2-methoxyphenol | 0.892 | phenylmethanethiol | 0.919 |
| 4-ethyl-2-methoxyphenol | 0.970 | prop-2-enyl prop-2-enoate | 0.916 |
| 4-ethylphenol | 0.954 | propanoic acid | 0.928 |
| 4-hydroxy-3-methoxybenzaldehyde | 0.914 | propyl decanoate | 0.911 |
| 4-methyl-2*H*-furan-5-one | 0.920 | tetradecanoic acid | 0.966 |
| 4-methylphenol | 0.902 | undecan-1-ol | 0.905 |
| 4-pentylphenol | 0.988 | undecan-2-ol | 0.887 |
| 4-propan-2-ylphenol | 0.913 | undecan-2-one | 0.924 |
| 4-propylphenol | 0.913 | *undecane* | 0.956 |

Table content does neither represent unequivocal compound identification nor a complete list of (aroma active) volatile compounds present in the whisky samples, all molecules should be considered as compound propositions and their presence in the samples has not been sufficiently verified. Furthermore, no reliable statements regarding chemical isomers can be made based on the present data. Compounds were considered as especially unlikely if they a) have been found with a confidence below 0.9 and were found in blank chromatograms (relating to the model whisky or the sample analysis) and have not been described in literature to occur in whisky to our knowledge; b) were detected with confidence below 0.9 and seen in blank chromatograms; c) have been detected in no more than 2 samples and with confidence below 0.9 (see table S6).

**Table S6** Alphabetical list of all molecules with especially low probability to be good matches detected in **any** of the 16 whisky samples (internal standards in italics) using a semi-automated compound detection tool and in-house database based on retention index and mass spectral data. Indication of mean confidence (mean match scores for compound detection) over all detected instances at highest relative peak intensity.^1^

| **IUPAC name** | **mean conf.** | **IUPAC name** | **mean conf.** |
| --- | --- | --- | --- |
| ^#^ (1*E*,4*E*,8*E*)-2,6,6,9-tetramethylcycloundeca-1,4,8-triene | 0.830 | *† 3,3-dimethylbutanoic acid | 0.844 |
| ^#^ (1*R*,2*R*,4*R*)-1,2,7,7-tetramethylbicyclo[2.2.1]heptan-2-ol | 0.847 | *† 4-ethyloctanoic acid | 0.853 |
| *† (1*R*,2*R*)-2-heptylcyclopropane-1-carboxylic acid | 0.877 | ^#^ 4-hexylphenol | 0.848 |
| ^#^ (1*S*,5*R*)-4-methyl-1-propan-2-ylbicyclo[3.1.0]hexan-3-one | 0.825 | †^#^ 4-hydroxy-2,5-dimethylfuran-3-one | 0.831 |
| † (2*E*)-3,7-dimethylocta-2,6-dien-1-ol | 0.876 | *† 4-methoxy-2-methylbutane-2-thiol | 0.897 |
| ^#^ (2*E*,4*E*)-hepta-2,4-dienal | 0.816 | *†^#^ 4-methylhexanoic acid | 0.809 |
| ^#^ (2*E*,4*E*)-nona-2,4-dienal | 0.885 | *† 4-methylnonanoic acid | 0.848 |
| ^#^ (2*E*,4*Z*)-deca-2,4-dienal | 0.824 | *† 4-methyloctanoic acid | 0.853 |
| ^#^ (2*E*,6*Z*)-nona-2,6-dien-1-ol | 0.866 | *† 4-methylpentanoic acid | 0.820 |
| ^#^ (2*E*,6*Z*)-nona-2,6-dienal | 0.837 | ^#^ 4-propan-2-ylbenzaldehyde | 0.842 |
| ^#^ (3-acetylphenyl) acetate | 0.857 | ^#^ 4,4,7a-trimethyl-6,7-dihydro-5*H*-1-benzofuran-2-one | 0.894 |
| *† (3-methyl-3-sulfanylbutyl) formate | 0.879 | ^#^ 5-heptyloxolan-2-one | 0.807 |
| *† (3*R*)-3-hydroxy-4,4-dimethyloxolan-2-one | 0.833 | † 5-hexyloxolan-2-one | 0.865 |
| *†^#^ (4*R*)-1-methyl-4-prop-1-en-2-ylcyclohexene | 0.863 | † 5-methyl-2-(2-sulfanylpropan-2-yl)cyclohexan-1-one | 0.840 |
| ^#^ (4*S*)-4-prop-1-en-2-ylcyclohexene-1-carbaldehyde | 0.814 | *† 5-methyl-2-propan-2-ylcyclohexan-1-ol | 0.825 |
| ^#^ (5*S*)-2-methyl-5-prop-1-en-2-ylcyclohex-2-en-1-one | 0.837 | ^#^ 5-methylhexanoic acid | 0.802 |
| † (*E*)-dec-2-enal | 0.883 | †^#^ 6-butyloxan-2-one | 0.865 |
| *† (*E*)-dodec-2-enal | 0.854 | ^#^ 6-methylhept-5-en-2-one | 0.851 |
| † (*E*)-hept-2-enal | 0.873 | *† 6-octyloxan-2-one | 0.839 |
| † (*E*)-non-2-enal | 0.839 | ^#^ 7-methyl-3-methylideneocta-1,6-diene | 0.805 |
| *† (*E*)-oct-2-enal | 0.892 | ^#^ [(1*R*,2*R*,4*R*)-1,7,7-trimethyl-2-bicyclo[2.2.1]heptanyl] prop-2-enoate | 0.853 |
| † (*Z*)-hex-3-en-1-ol | 0.827 | ^#^ acetaldehyde | 0.849 |
| *† (*Z*)-hexadec-9-enoic acid | 0.809 | ^#^ benzyl 2-bromoacetate | 0.857 |
| *† 1-(3,4-dihydro-2*H*-pyrrol-5-yl)ethanone | 0.882 | ^#^ bromo(dichloro)methane | 0.829 |
| ^#^ 1-chloro-2-methoxybenzene | 0.860 | ^#^ butan-2-ylbenzene | 0.811 |
| ^#^ 1-ethoxypropan-2-yl acetate | 0.896 | †^#^ butanoic acid | 0.893 |
| ^#^ 1-ethyl-2-methylbenzene | 0.890 | ^#^ butyl benzoate | 0.830 |
| ^#^ 1-fluoro-2-methoxybenzene | 0.815 | *† butyl prop-2-enoate | 0.854 |
| ^#^ 1-methoxynaphthalene | 0.854 | *† cyclohexanone | 0.854 |
| ^#^ 1-thiophen-2-ylethanone | 0.864 | ^#^ dec-1-ene | 0.820 |
| ^#^ 1,2-xylene | 0.877 | *† decan-2-one | 0.887 |
| *†^#^ 1,3-benzothiazole | 0.847 | † decanal | 0.892 |
| ^#^ 1,3-xylene | 0.815 | *† dibutyl decanedioate | 0.860 |
| ^#^ 1,5,9-trimethyl-12-propan-2-yl-15-oxabicyclo[10.2.1]pentadeca-5,9-dien-2-ol | 0.841 | †^#^ dimethyl pentanedioate | 0.839 |
| ^#^ 1*H*-pyrazin-2-one | 0.865 | † diphenylmethanone | 0.848 |
| *† 2-(2-methylpropoxy)ethanol | 0.832 | *† dodecanal | 0.860 |
| ^#^ 2-ethyl-3,5-dimethylpyrazine | 0.838 | *†^#^ ethyl (*Z*)-hexadec-9-enoate | 0.800 |
| ^#^ 2-ethyl-6-methylphenol | 0.872 | †^#^ ethyl (*Z*)-octadec-9-enoate | 0.849 |
| ^#^ 2-ethylhexyl acetate | 0.860 | ^#^ ethyl 2-methylpentanoate | 0.883 |
| ^#^ 2-hydroxy-3-methylcyclopent-2-en-1-one | 0.814 | ^#^ ethyl 2-sulfanylpropanoate | 0.802 |
| ^#^ 2-hydroxybenzaldehyde | 0.810 | ^#^ heptan-3-one | 0.851 |
| ^#^ 2-methoxy-4-[(*E*)-prop-1-enyl]phenol | 0.830 | † heptanal | 0.887 |
| ^#^ 2-methyl-1-phenylpropan-2-ol | 0.850 | ^#^ hex-1-en-3-one | 0.866 |
| † 2-methylbutanoic acid | 0.866 | † hexadecan-1-ol | 0.837 |
| † 2-methylbutyl acetate | 0.896 | † hexadecanoic acid | 0.842 |
| † 2-methylprop-2-enoic acid | 0.880 | *†^#^ hexane-3,4-dione | 0.899 |
| *† 2-methylpropanoic acid | 0.865 | *† hexyl benzoate | 0.885 |
| *†^#^ 2-octyloxirane | 0.810 | *† methyl (9*Z*,12*Z*,15*Z*)-octadeca-9,12,15-trienoate | 0.828 |
| *†^#^ 2-pentyl-2,3-dihydropyran-6-one | 0.808 | † methyl 2-(3-oxo-2-pentylcyclopentyl)acetate | 0.893 |
| ^#^ 2-phenylacetaldehyde | 0.870 | *† methyl 2-phenylacetate | 0.875 |
| *† 2-phenylethylbenzene | 0.855 | ^#^ methyl prop-2-enoate | 0.826 |
| ^#^ 2-propylphenol | 0.872 | ^#^ methyl undecanoate | 0.814 |
| ^#^ 2-sulfanylpropanoic acid | 0.812 | ^#^ n,n-dimethylmethanamine;hydrochloride | 0.817 |
| ^#^ 2,3-dimethylphenol | 0.860 | *† oct-1-en-3-one | 0.845 |
| ^#^ 2,3,6-trimethylphenol | 0.873 | † octanal | 0.872 |
| ^#^ 2,4,6-trimethylphenol | 0.892 | ^#^ oxolane-2,5-dione | 0.868 |
| ^#^ 2,5-dichlorophenol | 0.831 | *† pent-1-en-3-ol | 0.876 |
| *†^#^ 2,6-dimethoxyphenol | 0.802 | *† pentanal | 0.851 |
| *†^#^ 2,6-dimethyloct-7-en-2-ol | 0.891 | *† pentane-2,3-dione | 0.896 |
| ^#^ 3-chloro-4-propan-2-yloxybenzonitrile | 0.841 | *† pentanoic acid | 0.894 |
| †^#^ 3-chlorophenol | 0.837 | ^#^ pentyl benzoate | 0.864 |
| ^#^ 3-ethoxyphenol | 0.859 | *† phenyl benzoate | 0.840 |
| ^#^ 3-methylbut-2-en-1-ol | 0.837 | *†^#^ prop-2-enoic acid | 0.854 |
| ^#^ 3-methylbutyl benzoate | 0.886 | *† propyl benzoate | 0.889 |
| *†^#^ 3-methylheptan-4-one | 0.876 | *† tridecanal | 0.840 |
| † 3-methylphenol | 0.858 | *† undecanal | 0.876 |
| ^#^ 3-sulfanylbutan-2-one | 0.857 | † undecanoic acid | 0.872 |
| *† 3-sulfanylhexyl acetate | 0.823 |  |  |

Table content does neither represent unequivocal compound identification nor a complete list of (aroma active) volatile compounds present in the whisky samples, all molecules should be considered as compound propositions and their presence in the samples has not been sufficiently verified. Furthermore, no reliable statements regarding chemical isomers can be made based on the present data. Marked substances are especially unlikely to be good matches since they *have been found with a confidence below 0.9 and were found in blank chromatograms (relating to the model whisky or the sample analysis) and have not been described in literature to occur in whisky to our knowledge; † substances were detected with confidence below 0.9 and detected in blank chromatograms; ^#^ substances were detected in no more than 2 samples and with confidence below 0.9.
